# Anticarbamylated protein antibodies are associated with long-term disability and increased disease activity in patients with early inflammatory arthritis: results from the Norfolk Arthritis Register

**DOI:** 10.1136/annrheumdis-2015-207326

**Published:** 2015-10-06

**Authors:** Jennifer H Humphreys, Marije K Verheul, Anne Barton, Alexander J MacGregor, Mark Lunt, Rene EM Toes, Deborah PM Symmons, Leendert A Trouw, Suzanne MM Verstappen

**Affiliations:** 1 Arthritis Research UK Centre for Epidemiology, University of Manchester, Manchester, UK; 2 Department of Rheumatology, Leiden University Medical Center, Leiden, The Netherlands; 3 Department of Rheumatology, Norfolk and Norwich University Hospital, Norwich, UK; 4 NIHR Manchester Musculoskeletal Biomedical Research Unit, Manchester Academic Health Science Centre, Manchester, UK; 5 Arthritis Research UK Centre for Genetics and Genomics, University of Manchester, Manchester, UK

**Keywords:** Early Rheumatoid Arthritis, Disease Activity, Autoantibodies, Epidemiology, Rheumatoid Arthritis

## Abstract

**Objectives:**

Anticarbamylated protein (anti-CarP) antibodies are a novel family of autoantibodies recently identified in patients with inflammatory arthritis. The aim of this study was to investigate their association with long-term outcomes of disability and disease activity over 20 years’ follow-up in a cohort of patients with inflammatory polyarthritis (IP).

**Methods:**

Norfolk Arthritis Register recruited adults with recent-onset swelling of ≥2 joints for ≥4 weeks from 1990 to 2009. At baseline, Health Assessment Questionnaire (HAQ) and 28 joint disease activity scores (DAS28) were obtained, and C reactive protein, rheumatoid factor (RF), anticitrullinated protein antibodies (ACPA) and anti-CarP antibodies were measured. Further HAQ scores and DAS28 were obtained at regular intervals over 20 years. Generalised estimating equations were used to test the association between anti-CarP antibody status and longitudinal HAQ and DAS28 scores; adjusting for age, gender, smoking status, year of inclusion and ACPA status. Analyses were repeated in subgroups stratified by ACPA status. The relative association of RF, ACPA and anti-CarP antibodies with HAQ and DAS28 scores was investigated using a random effects model.

**Results:**

1995 patients were included; 1310 (66%) were female. Anti-CarP antibodies were significantly associated with more disability and higher disease activity, HAQ multivariate β-coefficient (95% CI) 0.12 (0.02 to 0.21), and these associations remained significant in the ACPA-negative subgroups. The associations of RF, ACPA and anti-CarP antibodies were found to be additive in the random effects model.

**Conclusions:**

Anti-CarP antibodies are associated with increased disability and higher disease activity in patients with IP. Our results suggest that measurement of anti-CarP antibodies may be useful in identifying ACPA-negative patients with worse long-term outcomes. Further, anti-CarP antibody status provided additional information about RF and ACPA.

## Background

Rheumatoid arthritis (RA) is a heterogeneous inflammatory arthritis, and individual patient outcomes can vary from mild to disabling and life limiting.^1^ ^2^ The presence or absence of autoantibodies provides important prognostic information to clinicians and patients. Rheumatoid factor (RF) and, in particular, anticitrullinated protein antibodies (ACPA) have been associated with more severe disease activity,^3^ ^4^ greater levels of disability^5^ and increased mortality.^6^ They also form part of the 2010 American College of Rheumatology (ACR)/European League Against Rheumatism (EULAR) classification criteria for RA.^7^ These criteria were developed with the aim of identifying patients with RA early in the natural history of the disease, using the initiation of disease-modifying antirheumatic drugs as their gold standard. Patients who lack ACPA and RF have been shown to be less likely to fulfil the 2010 RA criteria, although they may fulfil the older 1987 criteria.^8^ ^9^ Nevertheless, in clinical practice, there remains a subset of apparently seronegative patients who go on to experience high levels of disease activity and disability. If these patients could be distinguished from those patients with a milder disease course, they could benefit from early aggressive intervention.

Recently, a new group of autoantibodies, anticarbamylated protein (anti-CarP) antibodies, has been identified in the sera of patients with RA.^10^ These antibodies are directed against a post-translational modification of the amino acid lysine to homocitrulline in the presence of cyanate.^11^ They have been shown to predate the onset of symptoms,^12–14^ and may occur before or after the development of ACPA.^12^ Further, they have been shown to predict development of arthritis in patients with arthralgia.^15^ However, it is not yet known if they are associated with long-term disability and disease activity. In addition, it would be clinically relevant to understand the influence of anti-CarP antibody status in patients with and without the other autoantibodies (RF and ACPA), as well as how much prognostic information is contributed by each antibody.

As patients with anti-CarP antibodies may lack RF or ACPA, and therefore, be less likely to fulfil RA criteria, it is important to study a broad group of patients presenting with inflammatory polyarthritis (IP), which would include a subgroup that meet RA criteria. The aims of this study were (a) to describe the prevalence and co-occurrence of RF, ACPA and anti-CarP antibodies in patients with IP, (b) to investigate the relationship between anti-CarP antibody status and both disability and disease activity measured over time in patients presenting with IP, (c) to investigate these relationships in ACPA-positive and ACPA-negative subgroups and (d) to describe the additional predictive information provided by measuring these antibodies.

## Methods

### Patients and follow-up

Patients were included from the Norfolk Arthritis Register (NOAR). This cohort has been described previously^16^ Briefly, adults >16 years old with at least two swollen joints for at least 4 weeks in the former Norfolk Health Authority area were recruited between 1990 and 2009. Patients recruited from 1995 to 1999 were excluded from this study as they were not followed beyond 2 years. Patients were also excluded if no serum sample obtained within the first year after recruitment was available. The selection of patients for the analysis is shown in full in the online supplementary data file. At baseline, in NOAR, patients are assessed by a nurse who obtains demographic details, medication details and smoking history, and performs a 51 tender and swollen joint count. The patients complete the British version of the Health Assessment Questionnaire (HAQ).^17^ Patients in NOAR are followed up yearly for the first 3 years and then at 5, 7, 10, 15 and 20 years from baseline. Patients repeat the HAQ and the nurse assessment at each follow-up. Blood samples are taken at baseline and every 5 years thereafter, stored frozen and subsequently tested for RF (latex test), ACPA (Axis-Shield Diastat Anti-CCP kit) and C reactive protein (CRP) in Manchester, UK. The cut-offs for a positive test were set according to the manufacturers’ guidelines at >40 iu/L for RF, >5 iu/L for ACPA and >5 mg/L for CRP. The three-item disease activity score (DAS28-CRP)^18^ is calculated at baseline and every 5 years, and the 2010 ACR/EULAR criteria are applied retrospectively using baseline data. In 2013–2014, stored sera were sent to Leiden University Medical Center, The Netherlands, in a blinded fashion for measurement of anti-CarP antibodies using an in-house ELISA based on carbamylated fetal calf serum (FCS) as described before.^10^ Briefly, non-modified FCS and modified-FCS were coated on Nunc MaxiSorp plates (Thermo Scientific) overnight. After washing and blocking, the wells were incubated with serum. Bound human IgG was detected using rabbit antihuman IgG antibodies (Dako) and then horseradish peroxidase (HRP)-labelled goat antirabbit IgG antibody (Dako). Following final washings, HRP enzyme activity was visualised using 2,2'-azino-bis(3-ethylbenzothiazoline-6-sulphonic acid)^10^ NOAR is approved by the Norwich Local Research Ethics Committee, and all patients gave written consent.

### Statistical analysis

Differences in baseline disability (measured by the HAQ) and disease activity (measured by 28 joint disease activity score (DAS28)) between anti-CarP antibody positive and negative patients were evaluated using the Kruskal–Wallis test. Generalised estimating equations (GEE) were used to assess the association between anti-CarP antibody status and HAQ and DAS28 measured over time, allowing for the inclusion of patients with incomplete follow-up data. A time-interaction term was included to investigate any potential change in the relationship between baseline anti-CarP antibody status and HAQ or DAS28 scores. Univariate and subsequently multivariate models were constructed, adjusting for age, gender, smoking status (stratified as current, previous or never smoked), polynomials of disease duration (to better fit the outcome measures), year of recruitment to NOAR and ACPA status. The analyses were repeated in the ACPA-positive and ACPA-negative subgroups and in patients who did and did not meet the 2010 RA classification criteria at baseline, omitting the ACPA confounder variable. In addition, as DAS28 was only available every 5 years, sensitivity analyses were performed using the total swollen joint count as an alternative measure of disease activity over time.

The individual effects of RF, ACPA and anti-CarP antibodies were then investigated. For each of the two outcomes of interest, a random effects model was used to test the association with each antibody. A three-way interaction term was included to investigate potential interactions between the antibodies; the resulting β-coefficient for each antibody estimated the added effect of that antibody. The final model was also adjusted for age, gender, smoking status, disease duration and year of recruitment.

A proportion of patients had anti-CarP antibodies tested, but had missing data on some of the baseline covariates in the model (ACPA, CRP and smoking status; see table 1). To account for this, missing data were imputed using multiple imputation with chained equations, and a sensitivity analysis was performed in the imputed dataset. All analyses were performed using STATA V.12 software package (Stata, College Station, Texas, USA).

**Table 1:** Baseline demographic and disease characteristics

	Total cohort, n=1995	Patients with all antibodies tested, n=1476	Missing, n (% total cohort)
Female, n (%)	1310 (66)	983 (67)	0
Age at symptom onset (years), median (IQR)	55 (43–66)	54 (42–65)	0
Smoking status, n (%)			
Never	706/1982 (36)	535 (36)	13 (1%)
Previous	793/1982 (40)	585 (40)	
Current	483/1982 (24)	350 (24)	
Disease duration (weeks), median (IQR)	33 (17–69)	34 (17–70)	0
HAQ, median (IQR)	0.875 (0.375–1.5)	0.75 (0.25–1.5)	23 (1%)
DAS28, median (IQR)	3.81 (2.88–4.82)	3.79 (2.85–4.78)	362 (18%)
RF positive, n (%)	658/1895 (35)	463 (31)	100 (5%)
ACPA positive, n (%)	389/1487 (26)	385 (26)	508 (25%)
Anti-CarP antibody positive, n (%)	460 (23)	297 (20)	0
CRP (mg/L), median (IQR)	8.7 (2–19)	8 (2–18)	362 (18%)
Satisfy 2010 RA classification criteria* n (%)	1221 (61)	893 (61)	0
On DMARDs at baseline assessment n (%)	722 (36)	501 (34)	0

*At baseline.

ACPA, anticitrullinated protein antibodies; anti-CarP, anticarbamylated protein; CRP, C reactive protein; DAS28, 28 joint disease activity score; DMARDs, disease-modifying antirheumatic drugs; HAQ, Health Assessment Questionnaire; RA, rheumatoid arthritis; RF, rheumatoid factor.

## Results

**Figure 1:**
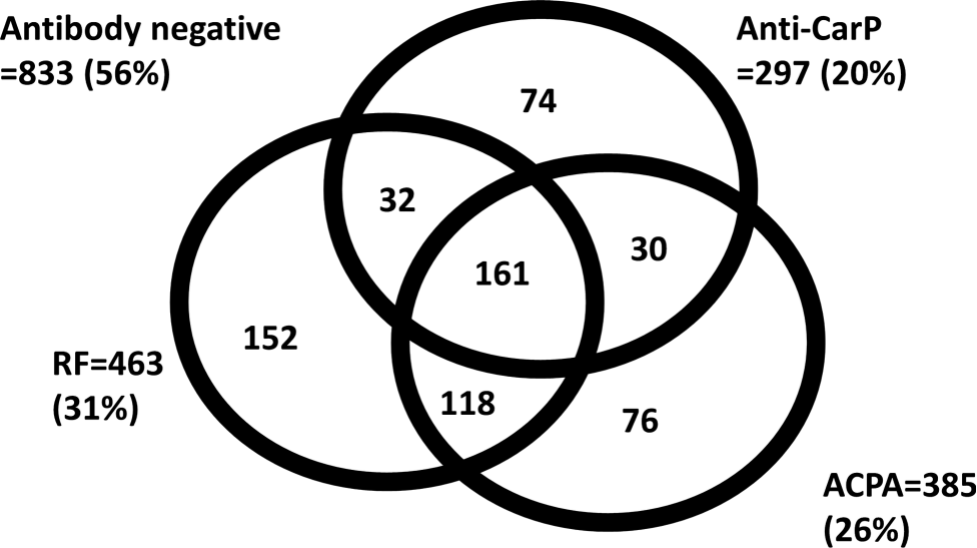
Distribution of antibodies in patients with IP who had all three antibodies tested. ACPA, anticitrullinated protein antibodies; anti-CarP, anticarbamylated protein; IP, inflammatory polyarthritis; RF, rheumatoid factor.

A total of 1995 patients with IP were included; 1310 (66%) were female, and median age at onset (IQR) was 55 (43–66) years. Four hundred and sixty (23%) patients were anti-CarP antibody positive, and 1221 (61%) fulfilled the 2010 ACR/EULAR classification criteria for RA at baseline. The median follow-up time (IQR) was 8 (5–12) years. A summary of the baseline characteristics is shown in table 1. Baseline characteristics of patients who fulfilled the 2010 RA criteria are shown in online supplementary table S1. A total of 1476 patients were tested for all three antibodies; of whom, 297 (20%) were anti-CarP antibody positive and 74 (5%) tested positive for only anti-CarP antibodies. The distribution of all antibody statuses is shown in figure 1.

Levels of disability at baseline differed between anti-CarP antibody positive and negative patients, respective median HAQ (IQR) 1.125 (0.5–1.75) and 0.875 (0.25–1.5), p<0.001. There were also differences in baseline DAS28 scores, respective median DAS28 (IQR) in the anti-CarP antibody positive and negative groups were 4.23 (3.19–5.31) and 3.73 (2.80–4.63), p<0.001.

**Figure 2:**
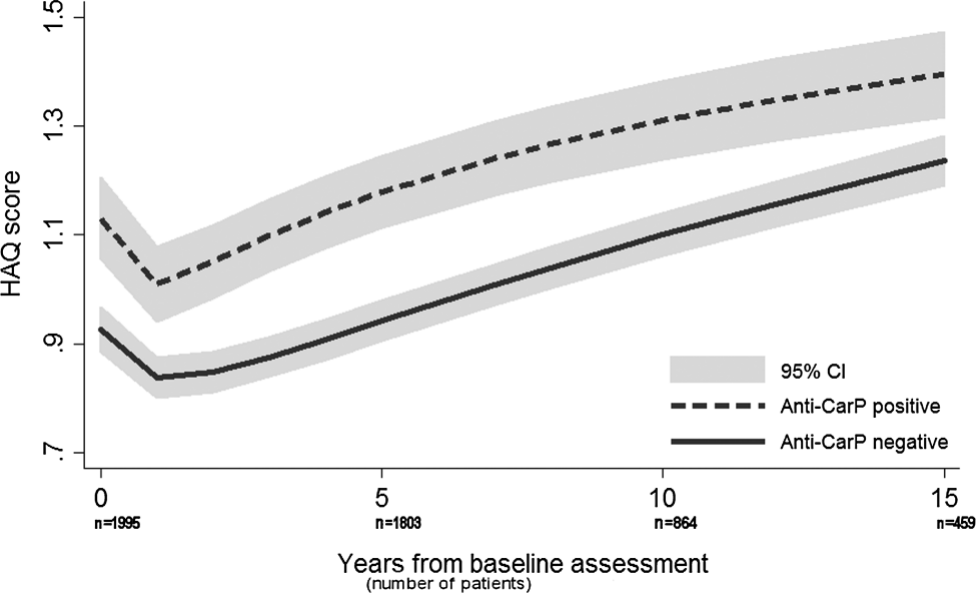
HAQ scores over time by anti-CarP antibody status (modelled by univariate GEE). Anti-CarP, anticarbamylated protein; GEE, generalised estimating equations; HAQ, Health Assessment Questionnaire.

In the GEE model, patients who were anti-CarP antibody positive were, on average, more disabled at baseline, and remained more disabled throughout follow-up compared with those who were negative (figure 2), unadjusted GEE β-coefficient (95% CI) 0.21 (0.14 to 0.29), and this remained significant in the multivariate analysis, including adjustment for ACPA status (table 2). Similarly, when DAS28 was the outcome of interest, anti-CarP antibody positive patients had, on average, higher levels of disease activity over time, unadjusted GEE β-coefficient 0.38 (0.25 to 0.50) (see online supplementary figure S2). This association persisted in the multivariate model. In the ACPA-negative subgroup, there was also a significant association between anti-CarP antibody positivity and HAQ. It should be noted that for both HAQ and DAS28, the multivariate β-coefficient (95% CI) were very similar between the ACPA-negative and ACPA-positive groups, and these estimates were not significantly different from each other. The interaction with time covariate was not statistically significant, meaning that the difference in HAQ scores between the average anti-CarP antibody positive patient and the average anti-CarP antibody negative patient did not increase or decrease over follow-up; this is displayed in figure 2. A time-interaction term was, therefore, not included in the final models.

**Table 2:** Association between anti-CarP antibody positivity and HAQ and DAS28

	Total cohort β (95% CI)	ACPA +ve β (95% CI)	ACPA −ve β (95% CI)
HAQ			
Univariate	0.21 (0.14 to 0.29)**	0.10 (−0.04 to 0.24)	0.18 (0.04 to 0.32)*
Multivariate†	0.12 (0.02 to 0.21)*	0.09 (−0.05 to 0.23)	0.14 (0.01 to 0.27)*
DAS28			
Univariate	0.38 (0.26 to 0.50)**	0.23 (0.01 to 0.46)*	0.11 (−0.11 to 0.34)
Multivariate*†	0.23 (0.07 to 0.39)*	0.25 (0.03 to 0.48)*	0.18 (−0.03 to 0.40)

*p<0.05.

**p<0.001.

†Adjusted for age, gender, smoking status, polynomials of disease duration and year of recruitment.

+ve, positive, −ve, negative; ACPA, anticitrullinated protein antibodies; anti-CarP, anticarbamylated protein; DAS28, 28 joint disease activity score; HAQ, Health Assessment Questionnaire.

In patients who fulfilled the 2010 RA criteria at baseline, anti-CarP antibody status was associated with DAS28, and there was a trend to statistical significance with HAQ, respective multivariate β-coefficients (95% CI) 0.18 (0.04 to 0.32) and 0.07 (−0.01 to 0.16) (see online supplementary table S2). Interestingly, there was a significant association with the HAQ among the group of patients who did not fulfil the 2010 RA criteria multivariate β-coefficient (95% CI) 0.19 (0.06 to 0.33). The sensitivity analysis with imputed missing covariates produced similar results (see online supplementary table S3), as did the sensitivity analysis with the swollen joint count as the outcome of interest (see online supplementary table S4).

In the model that assessed the relative contributions of ACPA, RF and anti-CarP antibodies to long-term disability, no interaction was found between the antibodies, and the R-squared value of the models were very similar with and without the interaction term. Therefore, the effect of each antibody could be considered to be additive rather than multiplicative. Both ACPA and anti-CarP antibodies were significantly associated with long-term disability, as measured by the HAQ, and had similar effect sizes, respective adjusted β-coefficient (95% CI) 0.12 (0.02 to 0.21) and 0.13 (0.03 to 0.21) (table 3). However, in the adjusted model, RF was not. In terms of disease activity over time, again, ACPA and anti-CarP antibodies were significantly associated with DAS28 score over time, and RF was not (table 3).

**Table 3:** Association between all autoantibodies and HAQ and DAS28

	Univariate β (95% CI)	Multivariate† β (95% CI)
HAQ		
ACPA	0.20 (0.12 to 0.28)**	0.12 (0.02 to 0.21)*
RF	0.12 (0.05 to 0.18)**	−0.03 (−0.12 to 0.05)
Anti-CarP antibodies	0.21 (0.14 to 0.29)**	0.13 (0.03 to 0.21)*
DAS28		
ACPA	0.36 (0.23 to 0.50)**	0.26 (0.09 to 0.43)**
RF	0.28 (0.17 to 0.39)**	−0.01 (−0.17 to 0.15)
Anti-CarP antibodies	0.38 (0.26 to 0.50)**	0.25 (0.09 to 0.42)**

*p<0.05.

**p<0.01.

†Adjusted for age, gender, smoking status, polynomials of disease duration and year of recruitment.

ACPA, anticitrullinated protein antibodies; anti-CarP, anticarbamylated protein; DAS28, 28 joint disease activity score; HAQ, Health Assessment Questionnaire; RF, rheumatoid factor.

## Discussion

This is the first study to investigate the associations between anti-CarP antibody status and long-term disease activity and disability in patients with IP. We have shown that patients with anti-CarP antibodies are more disabled, and have higher disease activity early in the disease and continue to have more functional disability and disease activity compared with anti-CarP antibody negative patients. We have also shown that the influence of anti-CarP antibody positivity is similar to that of ACPA when considering these outcomes and that measurement of the different autoantibodies provides additional information.

The majority of anti-CarP antibody positive patients in our study also demonstrated the presence of another antibody; however, there was a subset of patients who were only positive for anti-CarP antibodies. Of particular interest are the associations with poor outcomes in the ACPA-negative subgroup and the model adjusting for ACPA status. In general, ACPA-negative patients are considered to have a good prognosis.^3^ However, there is a small group who do poorly. For example, in studies of early arthritis cohorts, most patients who only fulfil the 1987 classification criteria for RA (characterised by the hallmarks of established RA such as radiological damage and nodulosis), and not the 2010 criteria (characterised by raised inflammatory markers and swollen/tender joint counts), are negative for RF and ACPA.^8^ ^9^ ^19^ Knowledge of anti-CarP antibody status in these patients, therefore, may be especially useful. In line with our results, other cohorts have demonstrated an association between anti-CarP antibody positivity and greater radiographic damage in patients with inflammatory arthritis, and the subgroup of these who are ACPA negative.^10^ ^14^ In our study, as well as stratifying patients with IP into ACPA-positive and ACPA-negative subgroups, we also adjusted for ACPA in the analyses of the whole cohort. This is because a number of studies have demonstrated that multiple autoantibodies can be accumulated in the preclinical phase of RA,^20^ ^21^ possibly via the mechanism of epitope spreading, and ACPA usually appears before RF.^22^ It, therefore, seemed reasonable to consider baseline ACPA status a potential confounder.

In this study, we have addressed, for the first time, the ‘added value’ of testing for anti-CarP antibodies. Recent studies in the literature have investigated the influence of the number of autoantibodies on disease outcomes.^6^ ^23^ Therefore, in addition to investigating the independent association of anti-CarP antibodies with disease outcomes, we wanted to address whether additional information is gained when RF and ACPA status is already known. It was interesting to note in this analysis that the coefficients for ACPA and anti-CarP antibodies were very similar. This suggests that, in terms of disability and disease activity over time, the impact of ACPA and anti-CarP antibodies are similar in patients with IP who test positive for these antibodies. Given that our results demonstrated an additive effect of each antibody, it may, therefore, be useful to test more than one antibody in clinical practice when trying to assess current and future disability and disease activity.

There are some limitations in our study. There are currently no commercial assays available to test for anti-CarP antibodies, which could restrict the clinical impact of these results. However, the assay based on the methods described by Shi *et al*^10^ has begun to be used more widely; to date, it has been employed by two independent groups.^24^ ^25^ In addition, a number of companies are developing routine assays to measure anti-CarP antibodies, which should become available in the near future.

It is important to acknowledge that the effect sizes demonstrated in this study do not meet some previously published ‘minimum clinically important difference’ (MCID) for the HAQ.^26^ However, these MCIDs were calculated and validated for use in clinical trials to test the effect of a specific treatment over a set period of time. Others have argued that the MCID estimates may be as low as 0.09 in observational studies.^27^ Our results certainly exceed this threshold. As mentioned above, the association of ACPA status with both HAQ and DAS28 demonstrated similar effect sizes to anti-CarP antibody status.

A larger proportion of patients recruited into NOAR were negative for all autoantibodies tested. This reflects the fact that patients with IP are a broad group, which includes a subset of patients with RA, and that the majority of patients are presenting early in their disease course. We have previously shown in this cohort that 75%–95% of patients recruited go on to satisfy the 1987 RA criteria.^28^ However, it is also important to note that the 2010 RA criteria do not identify all patients with inflammatory arthritis who subsequently have poor outcomes, and this is particularly seen in seronegative patients.^29^ It was interesting, therefore, that anti-CarP antibody positivity was associated with significantly higher HAQ scores in the subgroup of patients who did not satisfy the 2010 criteria at baseline. In these patients, anti-CarP antibodies may be a marker of those who will go on to develop RA.

We have not taken into account treatment in our analysis. However, we did include the year of registration in the multivariate models, which would account for changes in prescribing patterns since 1990. Importantly, anti-CarP antibodies and ACPA were tested on stored sera; therefore, the results were not known to the treating clinicians, and could not have influenced treatment decisions. ACPA status may have been available through testing in routine clinical practice; however, this would only apply to a small sample of NOAR patients seen by rheumatologists since 2009 when the test became widely available in Norfolk. In addition, as the anti-CarP antibody ELISA is a relatively new test, it is not yet clear whether prolonged storage of sera before testing may influence the results; adjustment for year of registration to NOAR will have taken some of this effect into account. The anti-CarP antibody positive patients had more active disease and more disability at baseline, and thus, may have had more intensive therapy, potentially introducing channelling bias. However, by not including the impact of treatment, we have biased our results towards the null hypothesis, and they are, therefore, likely to be an underestimate in terms of statistical significance. A further limitation is that we were not able to test the association between anti-CarP antibody status and radiological damage over time; this is due to the fact that not all patients in NOAR have radiographs, and, in those that do, they are taken at different follow-ups, depending on when the patient was recruited into the cohort. As a result, it would not be possible to describe the relationship over time in the same way as we have for disability and disease activity. Finally, there were some missing data on covariates in this study; multiple imputation with chained equations was used to allow inclusion of the whole sample in a sensitivity analysis, which gave similar results to the main findings.

This analysis has shown that anti-CarP antibodies may be an important additional family of antibodies in predicting long-term outcomes in patients with IP, and may be useful to test in addition to ACPA and RF.

## Supplementary Material

Web supplement
