# Supplementary material for: Anticarbamylated protein antibodies are associated with long-term disability and increased disease activity in patients with early inflammatory arthritis: results from the Norfolk Arthritis Register
Source: Ann Rheum Dis. 2015 Oct 6;75(6):1139–44. doi: 10.1136/annrheumdis-2015-207326 (PMC4893092; doi:10.1136/annrheumdis-2015-207326)
Supplement: Web supplement [file annrheumdis-2015-207326-s1.pdf]

Supplementary figure S1 NOAR patient flow chart

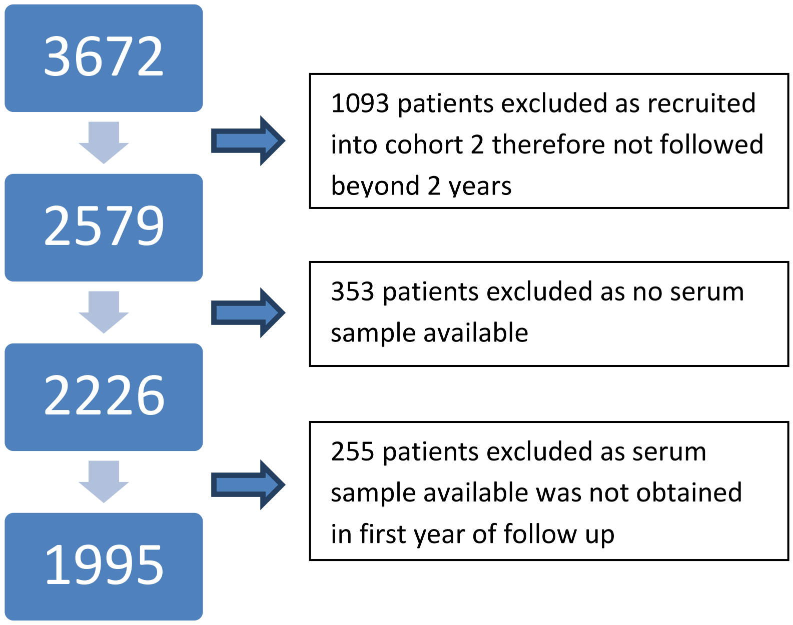

Supplementary table S1 Baseline characteristics of IP patients who fulfilled 2010 RA criteria at baseline

	**RA*** **n=1221**	**Missing** ***n (% RA)***
**Female *n (%)***	837(69)	0
**Age at symptom onset (years) *median (IQR)***	55 (45-66)	0
**Smoking status *n (%)***		7(1)
Never	408 (34)	
Previous	498 (41)	
Current	308 (25)	
**Disease duration (weeks) *median (IQR)***	33 (18-67)	0
**HAQ *median (IQR)***	1.125 (0.5-1.75)	14 (1)
**DAS28 *median (IQR)***	4.45 (3.69-5.37)	181 (15)
**RF positive *n (%)***	570 (48)	45 (4)
**ACPA positive *n (%)***	349 (39)	321 (26)
**Anti-CarP antibody positive *n (%)***	372 (30)	0
**CRP, (mg/L) *median (IQR)***	10.2 (3-23)	181 (15)
**On DMARDs at baseline assessment**	502 (41)	0

IQR, inter-quartile range; HAQ, Health Assessment Questionnaire; DAS28, 28 joint disease activity score; RF, rheumatoid factor; ACPA, anti-citrullinated protein antibodies; Anti-CarP, anti-carbamylated protein antibodies; CRP, C-reactive protein; RA, rheumatoid arthritis; DMARDs, disease modifying anti-rheumatic drugs

*satisfy the 2010 ACR/EULAR classification criteria at baseline assessment

Supplementary figure S2 Distribution of antibodies in patients who fulfil 2010 RA criteria at baseline

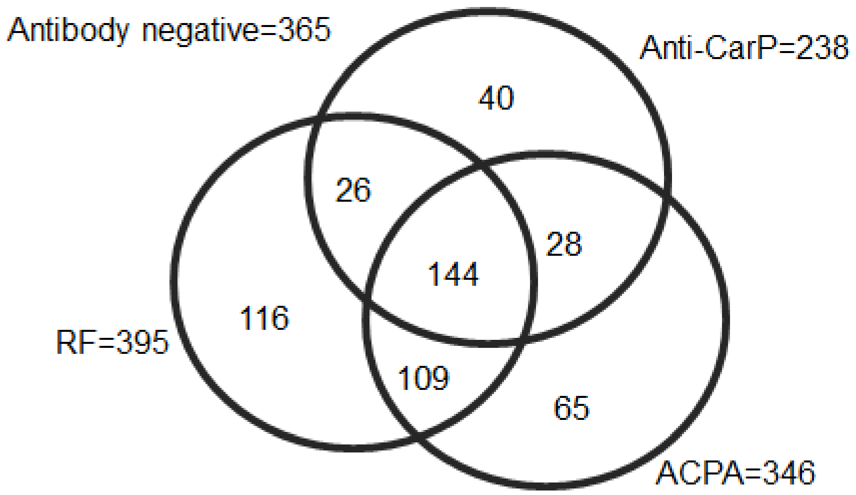

Supplementary figure S3 DAS28 scores over time by anti-CarP antibody status (modelled by univariate GEE)

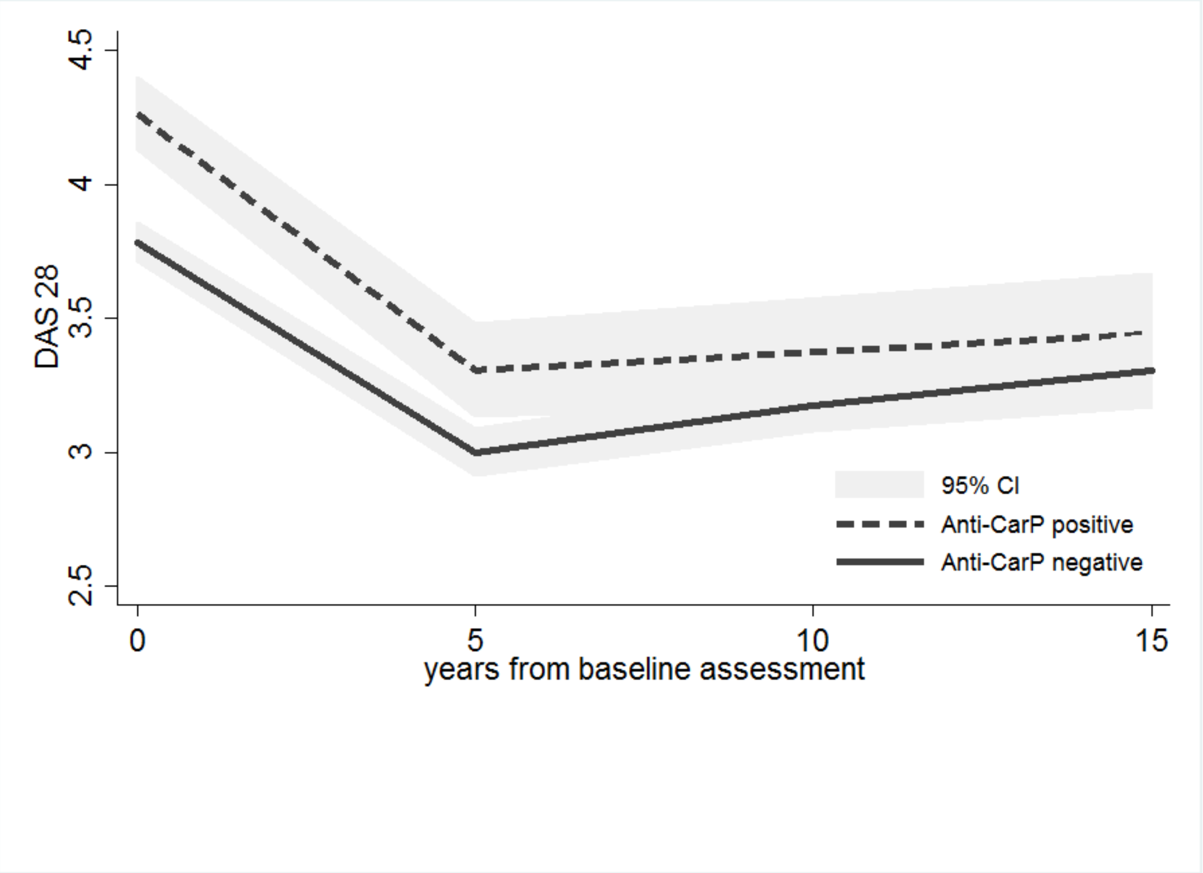

Supplementary figure S4 HAQ scores over time by ACPA status (modelled by univariate GEE)

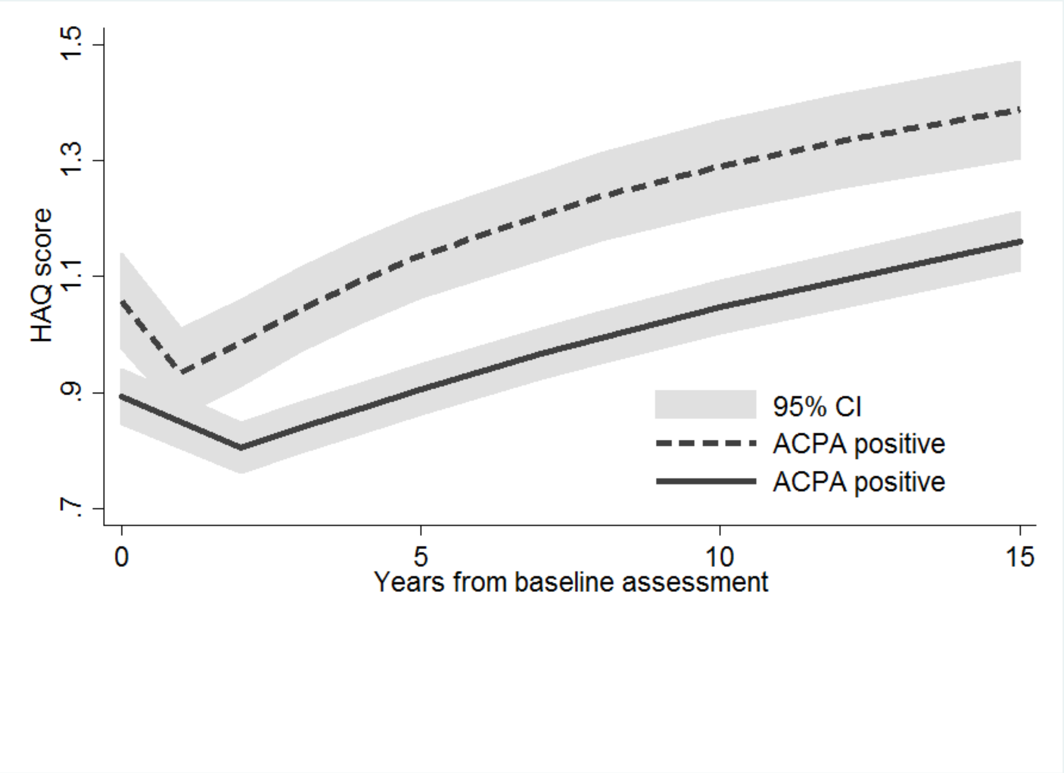

Supplementary figure S5 HAQ scores over time by RF status (modelled by univariate GEE)

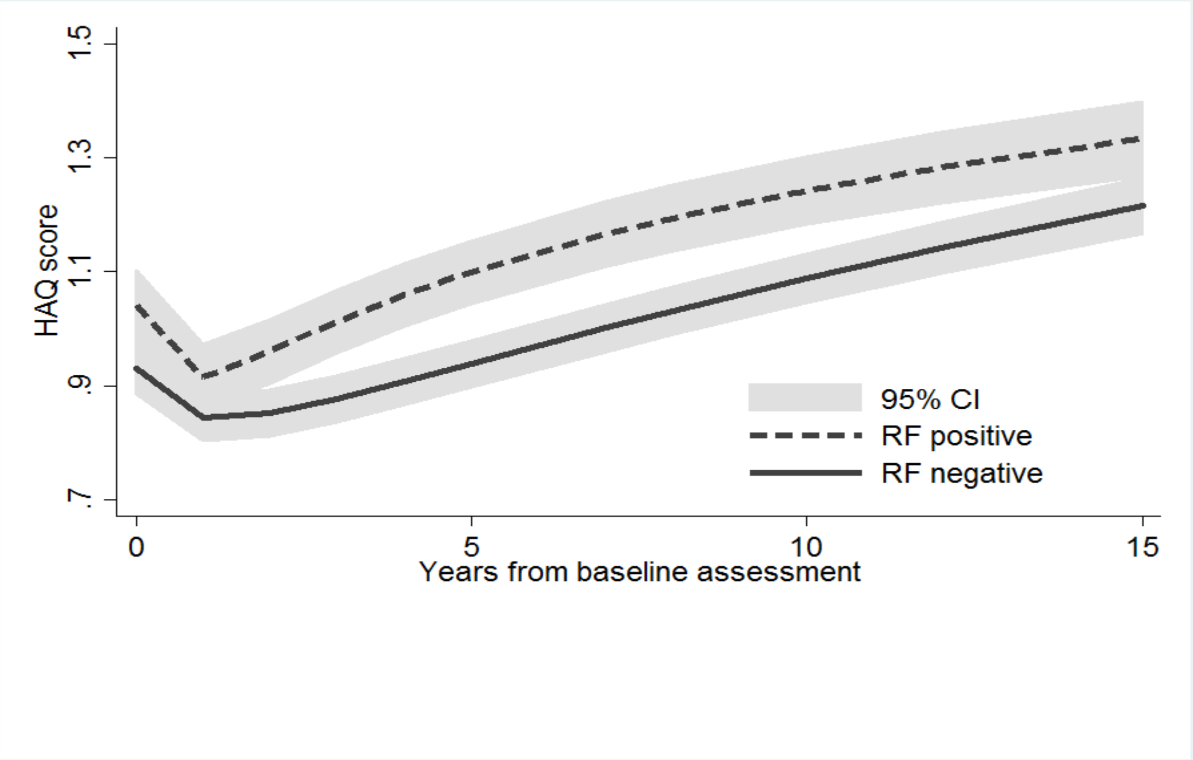

Supplementary table S2 GEE models including all covariates, with subgroups of patients stratified by 2010 RA classification criteria and by ACPA status

	**Total cohort**<br>*β (95% CI)*	**2010 RA +ve**<br>*β (95% CI)*	**2010 RA -ve**<br>*β (95% CI)*	**ACPA +ve**<br>*β (95% CI)*	**ACPA –ve**<br>*β (95% CI)*
**HAQ**					
Anti-CarP	0.13 (0.03,0.23)	0.07 (-0.01,0.16)	0.19(0.06,0.33)	0.10 (-0.04,0.25)	0.15 (0.02-0.29)
Disease duration	0.03 (0.02,0.03)	0.03 (0.02,0.03)	0.02(0.02,0.02)	0.03 (0.03,0.04)	0.02 (0.02-0.03)
ACPA	0.10 (0.01,0.19)	-	-	-	-
Age	0.02 (0.01,0.02)	0.02 (0.01,0.02)	0.01(0.01,0.01)	0.02 (0.01,0.02)	0.01 (0.01-0.02)
Female gender	0.36 (0.29,0.44)	0.36 (0.27,0.45)	0.25(0.16,0.34)	0.26 (0.11,0.42)	0.40 (0.31-0.49)
Smoking *never*	ref	ref	ref	Ref	ref
ex	0.04 (-0.04,0.13)	0.10 (0.00,0.19)	-0.04(-0.13,0.06)	-0.07 (-0.26,0.11)	0.08 (-0.01-0.17)
current	0.20 (0.10,0.30)	0.20 (0.09,0.31)	0.07(-0.04,0.19)	0.17 (-0.04,0.37)	0.20 (0.10-0.32)
Year of registration	0.01 (0.00,0.01)	0.00 (0.00,0.01)	0.01(0.00,0.02)	0.00 (-0.02,0.01)	0.01 (0.01-0.02)
**DAS28**					
Anti-CarP	0.23 (0.07,0.39)	0.18 (0.04,0.32)	0.01(-0.19,0.21)	0.25 (0.02,0.48)	0.22 (0.00-0.45)
Disease duration	-0.05 (-0.06,-0.04)	-0.09 (-0.10, -0.07)	0.01(0.00,0.02)	-0.06 (-0.08, -0.04)	-0.05 (-0.06- -0.04)
ACPA	0.27 (0.12,0.43)	-	-	-	-
Age	0.01 (0.00,0.01)	0.00 (0.00,0.01)	0.00(0.00,0.01)	0.00 (-0.01,0.01)	0.01 (0.00-0.01)
Female gender	0.49 (0.36,0.63)	0.26 (0.11,0.40)	0.32(0.17,0.46)	0.48 (0.23,0.72)	0.50 (0.35-0.66)
Smoking *never*	ref	ref	ref	Ref	ref
ex	0.13 (-0.04,0.27)	0.06 (-0.10,0.21)	-0.02(-0.18,0.13)	0.03 (-0.26,0.32)	0.16 (-0.01-0.32)
current	0.16 (-0.01,0.32)	0.10 (-0.07,0.27)	0.02(-0.16,0.19)	0.12 (-0.2,0.44)	0.17 (-0.02-0.36)
Year of registration	0.00 (-0.01,0.01)	-0.01 (-0.02,0.00)	0.02(0.01,0.03)	-0.03 (-0.05, -0.01)	0.02 (0.00-0.03)

2010 RA +ve, satisfied the 2010 ACR/EULAR classification criteria for RA at baseline assessment; 2010 RA -ve, did not satisfy the 2010 ACR/EULAR classification criteria for RA at baseline assessment; HAQ health assessment questionnaire; Anti-CarP, anti-carbamylated protein antibodies; ACPA, anti-citrullinated protein antibodies; DAS28, disease activity score

Supplementary table S3 Sensitivity analysis performed with imputed dataset*

	**Total cohort**<br>*β (95% CI)*	**2010 RA +ve**<br>*β (95% CI)*	**2010 RA -ve**<br>*β (95% CI)*
**HAQ**			
Anti-CarP	0.16(0.09,0.24)	0.06(-0.03,0.14)	0.19(0.05,0.32)
Age	0.01(0.01,0.02)	0.01(0.01,0.02)	0.01(0.01,0.01)
Sex	0.35(0.29,0.41)	0.34(0.26,0.42)	0.26(0.18,0.35)
Disease duration	-0.07(-0.09,-0.05)	-0.10(0.13,-0.07)	-0.02(-0.05,0.01)
Disease duration^2	0.02(0.01,0.02)	0.02(0.02,0.03)	0.008(0.001,0.015)
Disease duration^3	-0.001(-0.001,-0.001)	-0.001(-0.002,-0.001)	0.000(-0.001,0.000)
Smoking *never*	ref	Ref	Ref
current	0.12(0.05,0.19)	0.14(0.04,0.23)	0.05(-0.04,0.14)
previous	0.03(-0.02,0.09)	0.05(-0.03,0.13)	-0.01(-0.09,0.07)
Year of registration	0.007(0.002,0.012)	0.005(-0.001,0.011)	0.01(0.004,0.018)
ACPA	0.03(-0.02,0.07)	0.006(-0.053,0.065)	0.05(-0.02,0.13)
**DAS28**			
Anti-CarP	0.29 (0.18,0.40)	0.14(.02,0.27)	0.32(0.13,0.52)
Age	0.003(0.000,0.007)	0.002(-0.002,0.006)	0.002(-0.002,0.006)
Sex	0.38(0.29,0.47)	0.34(0.22,0.46)	0.23(0.12,0.35)
Disease duration	-0.62(-0.71,-0.54)	-0.85(-0.96,-0.74)	-0.28(-0.39,-0.17)
Disease duration^2	0.11(0.09,0.14)	0.15(0.12,0.17)	0.06(0.04,0.09)
Disease duration^3	-0.007(-0.009,-0.005)	-0.009(-0.012,-0.007)	-0.004(-0.007,-0.002)
Smoking *never*	ref	Ref	Ref
current	0.16(0.03,0.28)	0.15(-0.006,0.314)	0.06(-0.08,0.21)
previous	0.08(-0.03,0.19)	0.08(-0.05,0.21)	0.01(-0.13,0.15)
Year of registration	0.02(0.01,0.03)	0.02(0.01,0.03)	0.02(0.01,0.03)
ACPA	0.16(0.03,0.28)	-0.004(-0.147,0.140)	0.12(-0.09,0.32)

HAQ health assessment questionnaire; Anti-CarP, anti-carbamylated protein antibodies; ACPA, anti-citrullinated protein antibodies

*The imputed dataset was not used to create models in the ACPA subgroups due to the proportion of missing ACPA

Supplementary table S4 Sensitivity analyses performed with swollen joint count as the outcome of interest

	**Total cohort**<br>*β (95% CI)*	**2010 RA +ve**<br>*β (95% CI)*	**2010 RA -ve**<br>*β (95% CI)*	**ACPA +ve**<br>*β (95% CI)*	**ACPA –ve**<br>*β (95% CI)*
Anti-CarP	1.06 (1.43,2.25)	1.12 (0.61,1.64)	0.93 (0.41,1.45)	1.23 (0.33,2.13)	0.96 (0.25,1.66)
Disease duration	-0.27 (-0.29,-0.24)	-0.38 (-0.42,-0.35)	-0.07 (-0.09,-0.05)	-0.36 (-0.42,-0.30)	-0.23 (-0.25,-0.20)
ACPA	1.35 (0.85,1.84)	-	-	-	-
Age	0.02 (0.01,0.03)	0.00 (-0.01,0.02)	0.01 (0.00,0.02)	0.00 (-0.04,0.04)	0.02 (0.01,0.04)
Female gender	1.79 (1.36,2.21)	1.39 (0.86,1.93)	0.87 (0.52,1.22)	2.31 (1.36,3.26)	1.63 (1.17,2.09)
Smoking *never*	ref	ref	ref	Ref	ref
ex	-0.08 (-0.55,0.38)	-0.11 (-0.69,0.46)	-0.24 (-0.63,0.16)	-0.66 (-1.79,0.48)	0.10 (-0.39,0.59)
current	-0.37 (-0.90,0.16)	-0.42 (-1.06,0.23)	-0.28 (-0.73,0.17)	-0.64 (-1.86,0.58)	-0.30 (-0.87,0.28)
Year of registration	-0.12 (-0.15,-0.09)	-0.14 (-0.18,-0.10)	-0.03 (-0.06,-0.01)	-0.24 (-0.31,-0.16)	-0.08 (-0.12,-0.04)

2010 RA +ve, satisfied the 2010 ACR/EULAR classification criteria for RA at baseline assessment; 2010 RA -ve, did not satisfy the 2010 ACR/EULAR classification criteria for RA at baseline assessment; HAQ health assessment questionnaire; Anti-CarP, anti-carbamylated protein antibodies; ACPA, anti-citrullinated protein antibodies; DAS28, disease activity score
